# NR3C2-Related Transcriptome Profile and Clinical Outcome in Invasive Breast Carcinoma

**DOI:** 10.1155/2021/9025481

**Published:** 2021-01-28

**Authors:** Jianjun Lu, Fang Hu, Yingling Zhou

**Affiliations:** ^1^The Second School of Clinical Medicine, Southern Medical University, Guangzhou, Guangdong, China; ^2^Department of Cardiology, Guangdong Cardiovascular Institute, Guangdong General Hospital, Guangdong Academy of Medical Sciences, Guangzhou, China; ^3^Department of Medical Services, First Affiliated Hospital of Sun Yat-sen University, Guangzhou, Guangdong, China; ^4^Department of Child Health, Guangzhou Women and Children's Medical Center, Guangzhou Medical University, Guangzhou, Guangdong, China

## Abstract

**Background:**

Increasing evidence has indicated that the nuclear receptor subfamily 3 group C member 2 (NR3C2) may be associated with tumorigenesis and patient prognosis for certain types of tumors. However, the clinical significance of NR3C2 is unclear in invasive breast carcinoma (BRCA).

**Methods:**

We used bioinformatics to broadly investigate and obtain a deeper understanding of the prognostic significance between NR3C2 and BRCA. RNA-sequencing data and clinical information of patients with BRCA from the Cancer Genome Atlas database were collected for subsequent analysis. The diagnostic efficacy of NR3C2 was evaluated by calculating the receiver operating characteristic curve. The prognostic value of NR3C2 was evaluated by Kaplan-Meier analysis and Cox regression analysis for patients with BRCA. Moreover, the OSbrca database was used to validate NR3C2 as a prognostic biomarker for BRCA. Gene set enrichment analysis (GSEA) and tumor immune infiltration analysis were conducted to explore the molecular mechanism of NR3C2 in BRCA.

**Results:**

The expression level of NR3C2 in BRCA tissues decreased compared to that in normal breast tissues (*P* < 0.001). NR3C2 presented good diagnostic efficacy (AUC = 0.908). Moreover, the expression of NR3C2 was verified using the Oncomine database. High expression of NR3C2 was statistically associated with prolonged overall survival (HR = 0.65, 95% CI: 0.47-0.91, and *P* = 0.012), progression-free interval (HR = 0.68, 95% CI: 0.49-0.95, and *P* = 0.024), and disease-specific survival (HR = 0.57, 95% CI: 0.36-0.89, and *P* = 0.015) for patients with BRCA. Besides, the prognostic value of NR3C2 was verified by the OSbrca database. GSEA results suggested that enriched pathways included neuroactive ligand-receptor interaction, focal adhesion, and ECM-receptor interaction. NR3C2 expression was moderately correlated with mast cells and some T cell subsets in BRCA.

**Conclusion:**

NR3C2 is a potential prognostic biomarker that could help clinicians develop more appropriate treatment plans for individual patients with BRCA.

## 1. Introduction

Breast cancer is the leading cause of cancer deaths in women worldwide. In 2017, the global incidence of breast cancer rose to 1,960,681 cases, making it the third most common cancer in the world [1]. In developing and developed countries, the incidence and mortality of breast cancer are still increasing [2], and the survival rates of breast cancer in different countries are significantly different [3]. The increase in the global burden of breast cancer is mainly observed in countries with lower social development indexes [4]. In recent years, although the survival rate of breast cancer patients has improved, there is still a lack of early screening, detection, and cost-effective treatment [5]. As a supplement to clinical and pathological characteristics, prognostic biomarkers are increasingly urgently needed to help clinicians develop more appropriate treatment plans for individual patients with breast cancer.

Nuclear receptor subfamily 3 group C member 2 (NR3C2) encodes the adrenal cortex hormone receptor [6]. As a transcription-dependent factor, the adrenal cortex hormone receptor can bind to mineralocorticoid response elements to mediate the effect of aldosterone on the salt and water balance of restricted target cells. Abnormal expression of NR3C2 can lead to type I pseudo-hyperaldosteronism, hypertension in pregnancy, and chronic central serous chorioretinopathy [7–9]. Recent studies have shown that NR3C2 can inhibit the proliferation, invasion, and migration of certain tumor cells. In addition, NR3C2 has been reported as a tumor suppressor gene in clear cell renal cell carcinoma, pancreatic cancer, liver cancer, colon adenocarcinoma, oral squamous cell carcinoma, and glioblastoma cancer [10–15]. However, the clinical significance of NR3C2 in breast cancer remains unclear. Therefore, in this study, we used bioinformatics to broadly investigate and obtain a deeper understanding of the prognostic significance between NR3C2 and invasive breast carcinoma (BRCA).

## 2. Materials and Methods

### 2.1. RNA-Sequencing Data and Bioinformatics Analysis

We downloaded RNA-sequencing data and clinical information of patients with BRCA in HTSeq-FPKM format from the Cancer Genome Atlas (TCGA) (https://portal.gdc.cancer.gov/) and Genotype-Tissue Expression (GTEx) (https://commonfund.nih.gov/GTEx/) databases (*n* = 1065). Then, we converted HTSeq-FPKM format data to TPM (transcripts per million reads) format data and scaled these values with the following equation: log2(TPM + 1). Meanwhile, we downloaded RNA-sequencing data in TPM format from TCGA and GTEx for differential expression analysis of GTEx and pan-cancer analysis from UCSC Xena (https://xenabrowser.net/datapages/). In addition, this study complied with the publication guidelines described in TCGA database. Therefore, ethical approval and informed consent were not required (http://sancergenome.nih.gov/publications-/publicationguidelines).

### 2.2. Evaluation of Diagnostic Efficacy

The diagnostic efficacy of NR3C2 for BRCA was evaluated by calculating the receiver operating characteristic (ROC) curve. In the ROC curve, the abscissa is the false positive rate and the ordinate is the true positive rate. The closer the area under the curve (AUC) is to 1, the better the diagnostic efficacy is.

### 2.3. Validating NR3C2 Expression Using the Oncomine Database

The Oncomine database was used to confirm the expression patterns of NR3C2 in BRCA tissues (https://www.oncomine.org/resource/main.html) [16].

### 2.4. Survival Analysis

Univariate and multivariate Cox regression analyses were used to compare the prognostic value of NR3C2 expression and other clinical characteristics. Taking the median value of NR3C2 expression as a cut-off value, we performed survival analysis to calculate the overall survival (OS), progression-free interval (PFI), and disease-specific survival (DSS) in patients with BRCA. The Kaplan-Meier curve was used to evaluate the prognostic value of NR3C2 in BRCA. The prognosis data and the definitions of clinical survival outcome endpoints came from a study in which the authors analyzed the clinicopathological annotations of cancer patients in TCGA database and obtained TCGA clinical data resource, which can provide recommendations for the use of clinical endpoint indicators for 33 cancer types [17]. In their study, a standardized dataset called TCGA Pan-Cancer Clinical Data Resource (TCGA-CDR) was developed to ensure the proper use of the TCGA clinical dataset associated with genomic features, including four major clinical outcome endpoints: overall survival, progression-free interval, disease-free interval, and disease-specific survival.

The clinical survival outcome endpoints used in the present study were defined as follows. OS is the period from the date of diagnosis until the date of death from any cause. PFI is the period from the date of diagnosis until the date of the first occurrence of a new tumor event, which includes the progression of the disease, locoregional recurrence, distant metastasis, new primary tumor, or death with tumor. DSS was defined as death from the diagnosed cancer type, which has much greater relevance to cancer biology and therapeutic impact [17]. Moreover, the OSbrca database was used to verify the prognostic values of NR3C2 for BRCA (http://bioinfo.henu.edu.cn/BRCA/BRCAList.jsp) [18].

### 2.5. Statistical Analysis

All statistical analyses were performed using R software (3.6.2). The Wilcoxon signed rank sum test was used to analyze the difference of NR3C2 expression between tumor tissues and normal human tissues. The Wilcoxon signed rank sum test and logistic regression were used to analyze the relationship between clinical characteristics and NR3C2 expression. Moreover, the logistic regression method was used to analyze the relationship between clinicopathological characteristics of BRCA and a binary variable (high/low NR3C2 expression). Here, we took the binary variable (high/low NR3C2 expression) as the independent variable and a single clinicopathological feature as the dependent variable to calculate the odds ratio (OR). All hypothesis tests were two-sided tests, and a *P* value threshold of 0.05 was used in all tests to infer statistically significant changes.

### 2.6. GO (Gene Ontology) Enrichment Analysis and Gene Set Enrichment Analysis (GSEA)

The expression of NR3C2 was used to define phenotypes. In specific, the BRCA samples were split into high and low NR3C2 expression groups, with the median value of NR3C2 expression being used as the cut-off value. Subsequently, GO enrichment analysis and GSEA were performed to identify significantly enriched GO terms and pathways in the high NR3C2 expression phenotype using the clusterProfiler software package (3.6.2) of R software (http://www.bioconductor.org/packages/release/bioc/html/), respectively [19]. Genome arrangement was performed 1000 times in each analysis, and the adjusted *P* value (<0.05) was used to infer statistically significantly enriched terms.

### 2.7. Immune Infiltration Analysis

The previously reported marker genes of 24 immune cells were used to calculate the relative enrichment score of each immune cell. The infiltration of these immune cells in BRCA was then analyzed by the ssGSEA method, the relationship between NR3C2 expression and these immune cells was explored using the Spearman method, and the difference of immune infiltration between the high and low NR3C2 expression groups was tested by using the Wilcoxon signed rank sum test analysis. The 24 immune cells utilized for the aforementioned analysis included macrophages, eosinophils, neutrophils, natural killer (NK) cells, CD56bright NK cells, CD56dim NK cells, dendritic cells (DC), activated DC (aDC), immature DC (iDC), plasma cell-like DC (pDC), T cells, T helper cells, T follicular helper cells (Tfhs), CD8+ T cells, Th1 cells, Th2 cells, Th17 cells, Tregs, effector memory T cells (Tems), central memory CD4+ T cells (Tcms), and y*δ*T cells (Tgd) [20]. Besides, the correlation between NR3C2 expression and immune infiltration was explored by the TIMER2.0 database (http://timer.cistrome.org/) [21].

## 3. Results

### 3.1. Patient Characteristics

The characteristics of the patients are presented in Table 1. The dataset was collected from TCGA on May 15, 2020, including 1065 BRCA patients with gene expression data and clinical information. 110 out of 1065 BRCA patients had matched adjacent normal tissue samples. Besides, the RNA-sequencing data of normal breast tissues (*n* = 179) generated by the GTEx project was used to increase the sample size of normal tissues. The clinicopathological characteristics that were collected included age, race, pathologic stage, tumor status, histological type, PAM50, and HER2/ER/TP53 status. Regarding race, 6.15%, 18.34%, and 75.51% were Asian, Black or African American, and White, respectively. In terms of tumor stages, 180 (17.27%) were at Stage I, 606 (58.16%) were at Stage II, 238 (22.84%) were at Stage III, and 18 (1.73%) were at Stage IV. 539 (51.53%) of 1046 had lymph node metastasis. 20 (2.20%) of 304 had distant metastasis.

### 3.2. NR3C2 Expressions in BRCA Tissues

**Figure 1:**
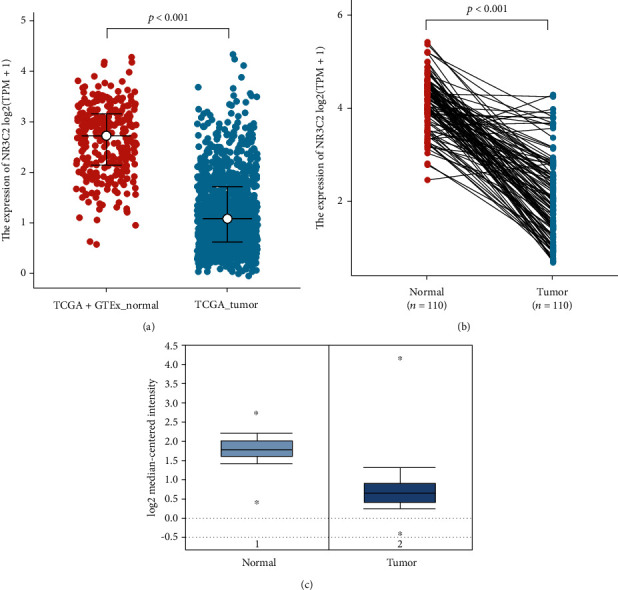
NR3C2 expressions in BRCA tissues. (a) NR3C2 expression in BRCA and normal human breast tissues (TCGA and GTEx databases). (b) NR3C2 expression in BRCA and matched normal breast tissues (TCGA database only). (c) The expression levels of NR3C2 in BRCA validated using the Oncomine database.

NR3C2 expressions in BRCA tissues were explored using the RNA-seq data from TCGA and GTEx databases. The difference of NR3C2 expressions between the tumor and normal human tissues in BRCA tissues is shown in Figure 1. The results suggested that NR3C2 expression significantly decreased in BRCA tissues compared to normal breast tissues in both TCGA+GTEx cohort (Figure 1(a)) and TCGA only cohort (Figure 1(b)). Moreover, NR3C2 expressions in pan-cancer were explored using the RNA-seq data of pan-cancer from TCGA and GTEx databases, as shown in Figure S1.

### 3.3. Validation of the NR3C2 Expression Using the Oncomine Database

In TCGA BRCA cohort, NR3C2 was highly expressed in normal breast tissues compared to BRCA. Next, we aimed to further confirm the expression patterns of NR3C2 in BRCA tissues in the Oncomine database. Consistent with our results in TCGA, the average expression levels of NR3C2 in normal breast tissues were significantly higher than those in BRCA tissues (Figure 1(c)).

### 3.4. NR3C2 Expression Was Correlated with Several Clinicopathological Characteristics

As shown in Figure S2, NR3C2 expression was correlated with several clinicopathological characteristics, including race (*P* < 0.001), tumor status (*P* = 0.024), histological type (*P* < 0.001), PAM50 (*P* < 0.001), HER2 status (*P* = 0.007), ER status (*P* = 0.046), and TP53 status (*P* < 0.001). Next, the logistic regression method was used to analyze the relationship between the clinicopathological characteristics and the classification of NR3C2 expression (high vs. low expression) in BRCA. In addition, we took the binary variable (high/low NR3C2 expression) as the independent variable and a single clinicopathological feature as the dependent variable to calculate the OR value. As shown in Table S1, NR3C2 expression was associated with clinicopathological characteristics, including race (odds ratio (OR): 0.46; 95% confidence interval (CI): 0.34-0.61; and *P* < 0.001), TP53 status (OR: 0.64; CI: 0.49-0.83; and *P* = 0.001), HER2 status (OR: 0.65; CI: 0.45-0.93; and *P* = 0.018), PAM50 (OR: 0.42; CI: 0.32-0.54; and *P* < 0.001), and histological type (OR: 0.42; CI: 0.30-0.58; and *P* < 0.001).

### 3.5. Diagnostic Efficacy of NR3C2 in BRCA

As we have known, the ROC curve can be used to evaluate the diagnostic efficacy of a diagnostic method. An AUC greater than 0.9 indicates that the diagnostic method has high accuracy. As shown in Figure S3, the AUC of NR3C2 was 0.908. This result suggested that NR3C2 expression presented a good ability in distinguishing BRCA tissues from normal human breast tissues. Moreover, the results showed that the best cut-off value, sensitivity, and specificity were 1.838, 0.777, and 0.887, respectively.

### 3.6. Prognostic Value of NR3C2 in BRCA

**Figure 2:**
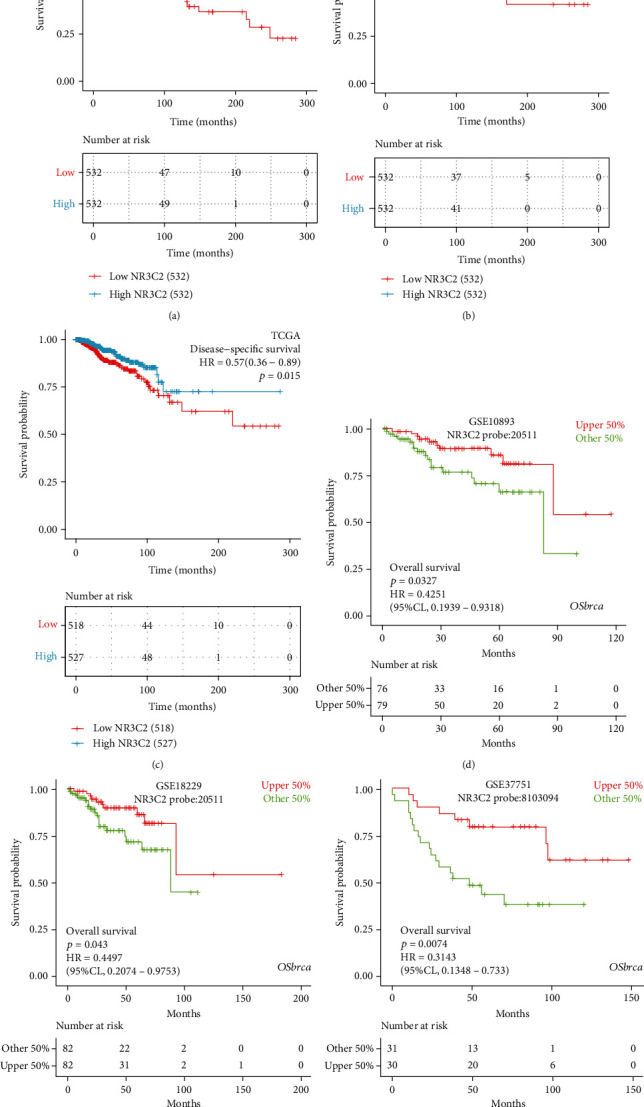
Prognostic value of NR3C2 for patients with BRCA was analyzed and validated using TCGA and GEO databases, respectively. As showed in the box diagram, the lower expression level of NR3C2 is associated with poor survival outcomes, including OS (a), PFI (b), and DSS (c). Besides, the prognostic value of NR3C2 on OS was verified with three BRCA cohorts from the GEO database using the OSbrca database (d–f). TCGA: The Cancer Genome Atlas; GEO: Gene Expression Omnibus; BRCA: invasive breast carcinoma; OS: overall survival; PFI: progression-free interval; DSS: disease-specific survival; GSE: GEO Series. ^∗^*P* < 0.05, ^∗∗^*P* < 0.01, and ^∗∗∗^*P* < 0.001. The prognosis data and the definitions of clinical survival outcome endpoints, including OS, PFI, DSS, used in Cox analysis came from an article published in the journal *Cell* in 2018 [17].

Kaplan-Meier analysis was performed to evaluate the prognostic value of NR3C2 expression in patients with BRCA. We divided the BRCA patients into high and low NR3C2 expression groups by taking the median value of NR3C2 expression as a cut-off value. As shown in Figure 2(a), the OS of the high NR3C2 expression group was significantly longer than that of the low NR3C2 expression group (hazard ratio (HR): 0.65; 95% confidence interval (CI): 0.47-0.91; and *P* = 0.012). Similarly, the PFI of the high NR3C2 expression group was significantly longer than that of the low NR3C2 expression group (HR: 0.68; 95% CI: 0.49-0.95; and *P* = 0.024; Figure 2(b)). The DSS of the high NR3C2 expression group was significantly higher than that of the low NR3C2 expression group as well (HR: 0.57; 95% CI: 0.36-0.89; and *P* = 0.015; Figure 2(c)).

Besides, univariate and multivariate Cox regression analyses were conducted to explore the associations between clinicopathological characteristics and the OS, PFI, and DSS of patients with BRCA. As shown in Table 2, the results of the multivariate analysis revealed that NR3C2 expression (HR: 0.446; CI: 0.268-0.743; and *P* = 0.002), pathologic stage (HR: 2.872; CI: 1.077-7.656; and *P* = 0.035), N stage (HR: 2.065; CI: 1.206-3.538; and *P* = 0.008), M stage (HR: 3.620; CI: 1.597-8.205; and *P* = 0.002), and radiation therapy (HR: 0.553; CI: 0.344-0.887; and *P* = 0.014) were independent factors of the OS for patients with BRCA. Similarly, the results of the multivariate analysis revealed that NR3C2 expression (HR: 0.558; CI: 0.371-0.838; and *P* = 0.005), N stage (HR: 1.846; CI: 1.155-2.951; and *P* = 0.010), M stage (HR: 5.342; CI: 2.753-10.365; and *P* < 0.001), and PR status (HR: 0.522; CI: 0.287-0.951; and *P* = 0.034) were independent factors of the PFI of patients with BRCA (Table S2). Moreover, NR3C2 expression (HR: 0.443; CI: 0.259-0.758; and *P* = 0.003), N stage (HR: 2.586; CI: 1.350-4.953; and *P* = 0.004), and M stage (HR: 6.995; CI: 3.358-14.571; and *P* < 0.001) were independent factors of the DSS for patients with BRCA (Table S3).

### 3.7. Validation of NR3C2 as a Prognostic Biomarker for BRCA

As shown in Figure 2, the prognostic value of NR3C2 was verified using the OSbrca database (BRCA cohorts from the GEO database were included). In the GEO BRCA cohorts, the OS of the high NR3C2 expression group was significantly longer than that of the low NR3C2 expression group as well (GSE10893: HR: 0.43; 95% CI: 0.19-0.93; and *P* = 0.033; Figure 2(d); GSE18229: HR: 0.45; 95% CI: 0.21-0.98; and *P* = 0.043; Figure 2(e); and GSE37751: HR: 0.31; 95% CI: 0.13-0.73; and *P* = 0.007; Figure 2(f)).

### 3.8. Enriched GO Terms and Pathways in the High NR3C2 Expression Phenotype

The BRCA samples were divided into high and low expression groups by using the median value of NR3C2 expression as a cut-off value. GO enrichment was conducted to identify important GO terms. GSEA was performed to identify significantly enriched pathways in the high NR3C2 expression phenotype group. As shown in Table S4, significantly enriched GO terms included muscle system process, regulation of membrane potential, receptor complex, contractile fiber, extracellular matrix structural constituent, and peptide receptor activity. Moreover, significantly enriched pathways included neuroactive ligand-receptor interaction, focal adhesion, ECM-receptor interaction, calcium signaling pathway, mismatch repair, protein export, homologous recombination, RNA polymerase, and fructose and mannose metabolism as depicted in Figure S4. Besides, the top 20 enriched KEGG pathways in the high NR3C2 expression phenotype group are presented in Table S5. These results indicated that NR3C2 may play an important role in the tumorigenesis and development of BRCA.

### 3.9. Correlation between NR3C2 Expression and Immune Infiltration

**Figure 3:**
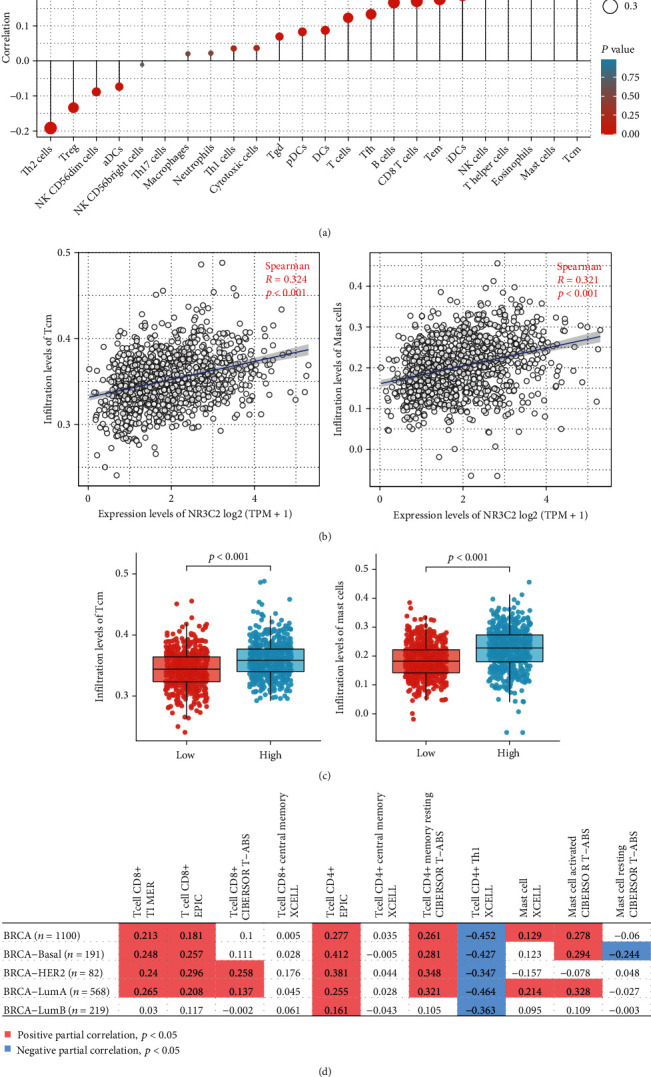
Associations between NR3C2 expression and immune infiltration in the tumor microenvironment in BRCA. (a) Correlation between the relative abundances of 24 immune cells and the NR3C2 expression level. The size of dots denotes the absolute value of the Spearman *r*. (b) Correlation between the relative enrichment score of immune cells (Tcm and mast cell) and the expression level (TPM) of NR3C2. (c) Comparison of immune infiltration levels of immune cells (Tcm and mast cell) between the high and low NR3C2 expression groups. (d) Correlation between NR3C2 expression and immune infiltration analyzed by the TIMER2.0 database. NK cells: natural killer cells; Tcm: central memory T cell.

As shown in Figures 3(a) and 3(b), the expression of NR3C2 was positively correlated with central memory T cells and mast cells (*r* > 0.3, *P* < 0.001). Moreover, the infiltration levels of central memory T cells and mast cells in the low NR3C2 expression phenotype were significantly lower than those in the high NR3C2 expression phenotype (Figure 3(c)). Subsequently, we analyzed the correlation between NR3C2 expression and immune infiltration using the TIMER2.0 database. The results suggested that the NR3C2 expression may be correlated with immune cells, including mast cells and some T cell subsets (Figure 3(d)).

## 4. Discussion

BRCA is known as a highly heterogeneous malignant tumor [22]. Currently, the diagnosis of BRCA mainly relies on the comprehensive evaluation of histopathological indicators such as pathologic staging, ER, PR, HER, Ki67, and PAM50 [23]. Because different molecular subtypes have different treatment sensitivities [24], the treatment of BRCA patients has undergone major changes in the past two decades, and the treatment for specific histological subtypes has improved the survival rate of BRCA patients. Therefore, in order to formulate more appropriate treatment plans for individual patients, pathological analysis and molecular subtypes should be identified. Therefore, reliable prognostic biomarkers of BRCA are urgently needed.

Screening out prognostic biomarkers is of great significance for individualized treatments and searching potential therapeutic targets for BRCA. NR3C2 has been reported as a tumor suppressor gene in certain tumors. Previous studies have shown that NR3C2 has prognostic value in certain tumors. For example, low NR3C2 expression was associated with poor prognosis in patients with nonmetastatic clear cell renal cell carcinoma [10] and colon adenocarcinoma [13]. Nevertheless, the clinical significance of NR3C2 in BRCA is unclear. In the present study, we explored the prognostic values of NR3C2 expression in BRCA. The results suggested that the expression of NR3C2 significantly decreased in BRCA. The NR3C2 expression profile was verified by independent BRCA cohorts from the Oncomine database. Besides, NR3C2 presented good diagnostic efficacy (ROC = 0.908) and prognostic value (OS; HR: 0.57; 95% CI: 0.36-0.89; and *P* = 0.015) in BRCA. Subsequently, the prognostic value of NR3C2 in BRCA was validated by using the OSbrca database. Therefore, NR3C2 is a potential prognostic biomarker for patients with BRCA. These results further enrich the content of prognostic biomarkers in BRCA.

Until now, there have been various types of diagnostic and/or prognostic biomarkers, including DNA methylation, noncoding RNA, mRNA, and protein in BRCA. For example, in terms of DNA methylation, TBCRC 005 has been reported as a prognostic indicator for patients with metastatic BRCA [25]. Besides, methylated circulating tumor DNA has been reported to be useful for monitoring clinical tumor response to neoadjuvant chemotherapy and postoperative recurrence for BRCA [26]. In terms of noncoding RNA, miR-222 may be related to endocrine therapy resistance and poor clinical outcomes in hormone receptor-positive breast cancer [27]. Circulating miRNAs including miR-16, miR-155, miR-195, miR-21, miR-222, and miR-373 can be used as biomarkers for the early diagnosis of BRCA [28, 29]. In terms of mRNA, gene signatures (GS) based on multiple mRNAs present good prognostic values in BRCA [30, 31]. In terms of protein, almost all newly diagnosed BRCA patients will be routinely measured for estrogen receptors (ERs) and progesterone receptors (PRs) before endocrine therapy. In addition, high expression of Ki67 is associated with more pathological complete remission events for neoadjuvant BRCA patients [32, 33]. Moreover, it is noted that different combinations of various serum tumor biomarkers, including carcinoembryonic antigen, cancer antigen 19-9, cancer antigen 125, cancer antigen 15-3, and tissue peptide-specific antigen, are of different diagnostic values in metastatic BRCA [34]. In addition to the above biomarkers, the ratios of neutrophils to lymphocytes and platelets to lymphocytes have also been reported as prognostic indicators of OS for BRCA [35, 36].

Previous studies have shown that the expression of NR3C2 can be downregulated by miR-135b-5p [11], miR-766 [12], miR-454 [14], and miR-1204 [15] in pancreatic cancer, liver cancer, oral squamous cell carcinoma, and glioblastoma, respectively, thereby promoting the malignant phenotype of tumor cells in above cancers. In our study, the GSEA results showed that the important enrichment pathways in the high NR3C2 expression phenotype group included neuroactive ligand-receptor interaction, focal adhesion, ECM-receptor interaction, calcium signaling pathway, and mismatch repair. These results suggest that NR3C2 may play an important role in tumorigenesis and development of certain cancers including BRCA.

Malignant tumors including BRCA are usually infiltrated by immune cells. Previous studies have shown that cellular immune infiltration from innate and adaptive immune responses in tumor tissues is related to the prognosis of patients [37]. Therefore, tumor immune infiltration can be used to evaluate the prognosis for tumor patients [38]. And immune cells and immunomodulatory biomarkers may be potential targets for enhancing the sensitivity of immunosuppressive tumors to various treatments [39].

The immune checkpoint inhibitor (ICI) is one of the important methods for the treatment of certain cancers. However, due to the lack of infiltrating immune cells, many BRCA patients respond weakly to immune checkpoint inhibitors. Therefore, screening infiltrating immune cells in breast cancer has important clinical value for combined ICI treatment of BRCA.

A recent study reported that the expression of chemokine CXC receptors (CXCR), which played a key role in leukocyte infiltration, was closely related to tumor-infiltrating lymphocytes (TILs) and immune checkpoints in BRCA. Further analysis results showed that high mRNA expressions of CXCR3/4/5/6 were significantly related to the recurrence-free survival of BRCA patients [40]. Moreover, previous research suggested that tumor-infiltrating lymphocytes (TILs) are an important part of the BRCA immune response, and increased expression of interstitial TILs is significantly correlated with the expression of cancer stem cell markers [41, 42]. In addition, tumor-associated macrophages (TAMs) can increase the expression of uPAR and Ki67 in tumor cells, thereby affecting the prognosis of BRCA patients [43]. In addition, CD68-, CD163-, and CD204-positive TAMs can be used to assess the progress of BRCA [44, 45]. The results of this study suggested that the infiltration levels of central memory T cells and mast cells were positively correlated with the expression of NR3C2 (*r* > 0.3, *P* < 0.01). This suggests that NR3C2 may be associated with immune infiltration in BRCA tissues. Moreover, the results of subsequent validation analysis using the TIMER2.0 database suggested that the NR3C2 expression was moderately correlated with immune cells, including mast cells and some T cell subsets. These results may provide some basis for exploring the application of ICI in the treatment of BRCA.

However, there were some limitations to this study. For example, all the data included in the study came from online databases. More biological and clinical experiments are needed to verify the prognostic values of NR3C2. In conclusion, BRCA as we have known is one of the most important threats to women's health now. So early detection and individualized treatment are of great importance to improve the prognosis of patients with BRCA. Prognostic biomarkers of BRCA are an important supplement to traditional indicators such as TNM staging for evaluating the prognosis of patients. In our study, the NR3C2 expression profile and prognostic values of NR3C2 were analyzed. The results suggest that high NR3C2 expression was associated with prolonged OS, PFI, and DSS in patients with BRCA. Taken together, NR3C2 is a potential prognostic biomarker that could help clinicians develop more appropriate treatment plans for individual patients with BRCA.

## Figures and Tables

**Table 1:** Clinicopathological characteristics of high and low NR3C2 expression groups.

Characteristics	Levels	Low expression of NR3C2	High expression of NR3C2	*P* value
*n*		533	532	
Age (mean (SD))		59.02 (13.86)	57.68 (12.46)	0.097
Race (%)	Asian	43 (8.9%)	17 (3.4%)	<0.001
	Black or African American	110 (22.8%)	69 (14.0%)	
	White	330 (68.3%)	407 (82.6%)	

Menopause status (%)	Peri	20 (4.2%)	19 (4.0%)	0.980
	Post	346 (72.5%)	347 (72.4%)	
	Pre	111 (23.3%)	113 (23.6%)	

T stage (%)	T1	130 (24.5%)	145 (27.3%)	0.009
	T2	315 (59.3%)	300 (56.5%)	
	T3	60 (11.3%)	77 (14.5%)	
	T4	26 (4.9%)	9 (1.7%)	

N stage (%)	N0	261 (49.9%)	246 (47.0%)	0.579
	N1	168 (32.1%)	181 (34.6%)	
	N2	54 (10.3%)	62 (11.9%)	
	N3	40 (7.6%)	34 (6.5%)	

M stage (%)	M0	442 (97.4%)	447 (98.2%)	0.494
	M1	12 (2.6%)	8 (1.8%)	

Pathologic stage (%)	Stage I	93 (18.0%)	87 (16.6%)	0.869
	Stage II	300 (57.9%)	306 (58.4%)	
	Stage III	115 (22.2%)	123 (23.5%)	
	Stage IV	10 (1.9%)	8 (1.5%)	

PR status (%)	Negative	178 (35.2%)	160 (31.6%)	0.257
	Positive	328 (64.8%)	346 (68.4%)	
ER status (%)	Negative	131 (25.8%)	106 (20.9%)	0.072
	Positive	376 (74.2%)	402 (79.1%)	

HER2 status (%)	Negative	255 (73.9%)	293 (81.4%)	0.022
	Positive	90 (26.1%)	67 (18.6%)	

PAM50 (%)	Basal	105 (19.7%)	85 (16.0%)	<0.001
	HER2	55 (10.3%)	27 (5.1%)	
	LumA	228 (42.8%)	323 (60.7%)	
	LumB	138 (25.9%)	64 (12.0%)	
	Normal	7 (1.3%)	33 (6.2%)	

Histological type (%)	Infiltrating ductal carcinoma	406 (86.0%)	351 (72.1%)	<0.001
	Infiltrating lobular carcinoma	66 (14.0%)	136 (27.9%)	

Tumor status (%)	Tumor free	441 (86.3%)	469 (90.2%)	0.065
	With tumor	70 (13.7%)	51 (9.8%)	

TP53 status (%)	Mut	198 (39.9%)	137 (29.8%)	0.001
	WT	298 (60.1%)	323 (70.2%)	

PIK3CA status (%)	Mut	163 (32.9%)	151 (32.8%)	1.000
	WT	333 (67.1%)	309 (67.2%)	

**Table 2:** Univariate/multivariate Cox regression analyses on the overall survival in BRCA.

Characteristics	Total (*n*)	HR (95% CI)	*P* value	HR (95% CI)	*P* value
		Univariate analysis	Univariate analysis	Multivariate analysis	Multivariate analysis
Age (>60 vs. ≤60)	1064	2.036 (1.468-2.822)	<0.001	2.203 (1.197-4.055)	0.011
Anatomic neoplasm subdivisions (right vs. left)	1064	0.776 (0.559-1.077)	0.130		
Menopause status (Pre&Peri vs. Post)	955	0.416 (0.250-0.692)	<0.001	0.899 (0.429-1.885)	0.777
Pathologic stage (Stage II&Stage III&Stage IV vs. Stage I)	1041	2.141 (1.270-3.609)	0.004	2.872 (1.077-7.656)	0.035
T stage (T2&T3&T4 vs. T1)	1061	1.435 (0.973-2.116)	0.069		
N stage (N1&N2&N3 vs. N0)	1045	2.145 (1.497-3.073)	<0.001	2.065 (1.206-3.538)	0.008
M stage (M1 vs. M0)	909	4.327 (2.508-7.465)	<0.001	3.620 (1.597-8.205)	0.002
Radiation therapy (yes vs. no)	971	0.558 (0.381-0.819)	0.003	0.553 (0.344-0.887)	0.014
TP53 status (Mut vs. WT)	955	1.218 (0.858-1.730)	0.269		
PIK3CA status (Mut vs. WT)	955	1.015 (0.696-1.479)	0.938		
PAM50 (LumB&HER2&Basal vs. LumA)	1024	1.547 (1.109-2.158)	0.010	1.390 (0.851-2.272)	0.188
Race (Black or African American&Asian vs. White)	975	1.136 (0.765-1.687)	0.526		
PR status (positive vs. negative)	1011	0.762 (0.541-1.074)	0.120		
ER status (positive vs. negative)	1014	0.704 (0.487-1.017)	0.062		
HER2 status (positive vs. negative)	705	1.611 (0.981-2.644)	0.059		
Histological type (infiltrating ductal carcinoma vs. infiltrating lobular carcinoma)	959	1.162 (0.738-1.830)	0.516		
NR3C2 (high vs. low)	1064	0.653 (0.468-0.911)	0.012	0.446 (0.268-0.743)	0.002

## Data Availability

The datasets analyzed in the current study are available in TCGA (https://portal.gdc.cancer.gov/) and GTEx (https://commonfund.nih.gov/GTEx/) databases.

## Supplementary Materials

Supplementary Materials Figure S1: human NR3C2 expressions in pan-cancer (TCGA and GTEx databases). ACC: adrenocortical carcinoma; BLCA: bladder urothelial carcinoma; BRCA: invasive breast carcinoma; CESC: cervical squamous cell carcinoma and adenocarcinoma; CHOL: cholangiocarcinoma; COAD: colon cancer; ESCA: esophageal carcinoma; GBM: multiform glioma; HNSC: head and neck squamous cell carcinoma; KICH: renal chromophobe cell carcinoma; KIRC: renal clear cell carcinoma; KIRP: renal papillary cell carcinoma; LAML: acute myeloid leukemia; LGG: brain low-grade glioma; LIHC: hepatocellular carcinoma; LUAD: lung adenocarcinoma; LUSC: lung squamous cell carcinoma; OV: ovarian serous cystadenocarcinoma; PAAD: pancreatic cancer; PCPG: pheochromocytoma and paraganglioma; PRAD: prostate cancer; READ: rectal adenocarcinoma; SARC: sarcoma; SKCM: cutaneous melanoma; TGCT: testicular cancer; THCA: thyroid cancer; THYM: thymic cancer; UCEC: endometrial cancer; UCS: uterine sarcoma; TCGA: The Cancer Genome Atlas; GTEx: Genotype-Tissue Expression. ^∗^*P* < 0.05, ^∗∗^*P* < 0.01, and ^∗∗∗^*P* < 0.001. Figure S2: NR3C2 expression in subgroups of patients with BRCA, stratified based on various clinicopathological characteristics, including age, race, pathologic stage, tumor status, histological type, PAM50, and HER2/ER/TP53 status in TCGA database (A–I). ^∗^*P* < 0.05, ^∗∗^*P* < 0.01, and ^∗∗∗^*P* < 0.001. Figure S3: diagnostic efficacy of NR3C2 in invasive breast carcinoma evaluated by ROC curves. ROC: receiver operating characteristic. Figure S4: enrichment plots from the gene set enrichment analysis (GSEA) tool. Several pathways were differentially enriched in the high NR3C2 expression phenotype of BRCA, including the neuroactive ligand-receptor interaction (A), focal adhesion (B), ECM-receptor interaction (C), calcium signaling pathway (D), mismatch repair (E), protein export (F), homologous recombination (G), RNA polymerase (H), and fructose and mannose metabolism (I). The GSEA software was used to calculate the enrichment levels. GSEA: gene set enrichment analysis; ES: enrichment score; NES: normalized ES; ADJP-val: adjusted *P* value. Table S1: NR3C2 expression was associated with several clinicopathological characteristics. Table S2: univariate/multivariate Cox regression analysis on the progression-free survival in BRCA. Table S3: univariate/multivariate Cox regression analysis on the disease-specific survival in BRCA. Table S4: several significantly enriched GO terms in the high phenotype group. Table S5: top 20 KEGG pathways enriched in the high phenotype group.
