# Supplementary material for: NR3C2-Related Transcriptome Profile and Clinical Outcome in Invasive Breast Carcinoma
Source: Biomed Res Int. 2021 Jan 28;2021:9025481. doi: 10.1155/2021/9025481 (PMC7867450; doi:10.1155/2021/9025481)
Supplement: Supplementary Materials — Figure S1: human NR3C2 expressions in pan-cancer (TCGA and GTEx databases). ACC: adrenocortical carcinoma; BLCA: bladder urothelial carcinoma; BRCA: invasive breast carcinoma; CESC: cervical squamous cell carcinoma and adenocarcinoma; CHOL: cholangiocarcinoma; COAD: colon cancer; ESCA: esophageal carcinoma; GBM: multiform glioma; HNSC: head and neck squamous cell carcinoma; KICH: renal chromophobe cell carcinoma; KIRC: renal clear cell carcinoma; KIRP: renal papillary cell carcinoma; LAML: acute myeloid leukemia; LGG: brain low-grade glioma; LIHC: hepatocellular carcinoma; LUAD: lung adenocarcinoma; LUSC: lung squamous cell carcinoma; OV: ovarian serous cystadenocarcinoma; PAAD: pancreatic cancer; PCPG: pheochromocytoma and paraganglioma; PRAD: prostate cancer; READ: rectal adenocarcinoma; SARC: sarcoma; SKCM: cutaneous melanoma; TGCT: testicular cancer; THCA: thyroid cancer; THYM: thymic cancer; UCEC: endometrial cancer; UCS: uterine sarcoma; TCGA: The Cancer Genome Atlas; GTEx: Genotype-Tissue Expression. ∗P < 0.05, ∗∗P < 0.01, and ∗∗∗P < 0.001. Figure S2: NR3C2 expression in subgroups of patients with BRCA, stratified based on various clinicopathological characteristics, including age, race, pathologic stage, tumor status, histological type, PAM50, and HER2/ER/TP53 status in TCGA database (A–I). ∗P < 0.05, ∗∗P < 0.01, and ∗∗∗P < 0.001. Figure S3: diagnostic efficacy of NR3C2 in invasive breast carcinoma evaluated by ROC curves. ROC: receiver operating characteristic. Figure S4: enrichment plots from the gene set enrichment analysis (GSEA) tool. Several pathways were differentially enriched in the high NR3C2 expression phenotype of BRCA, including the neuroactive ligand-receptor interaction (A), focal adhesion (B), ECM-receptor interaction (C), calcium signaling pathway (D), mismatch repair (E), protein export (F), homologous recombination (G), RNA polymerase (H), and fructose and mannose metabolism (I). The GSEA software was used to calculate the enrichment level [file 9025481.f1.docx]

# Supplementary Figures


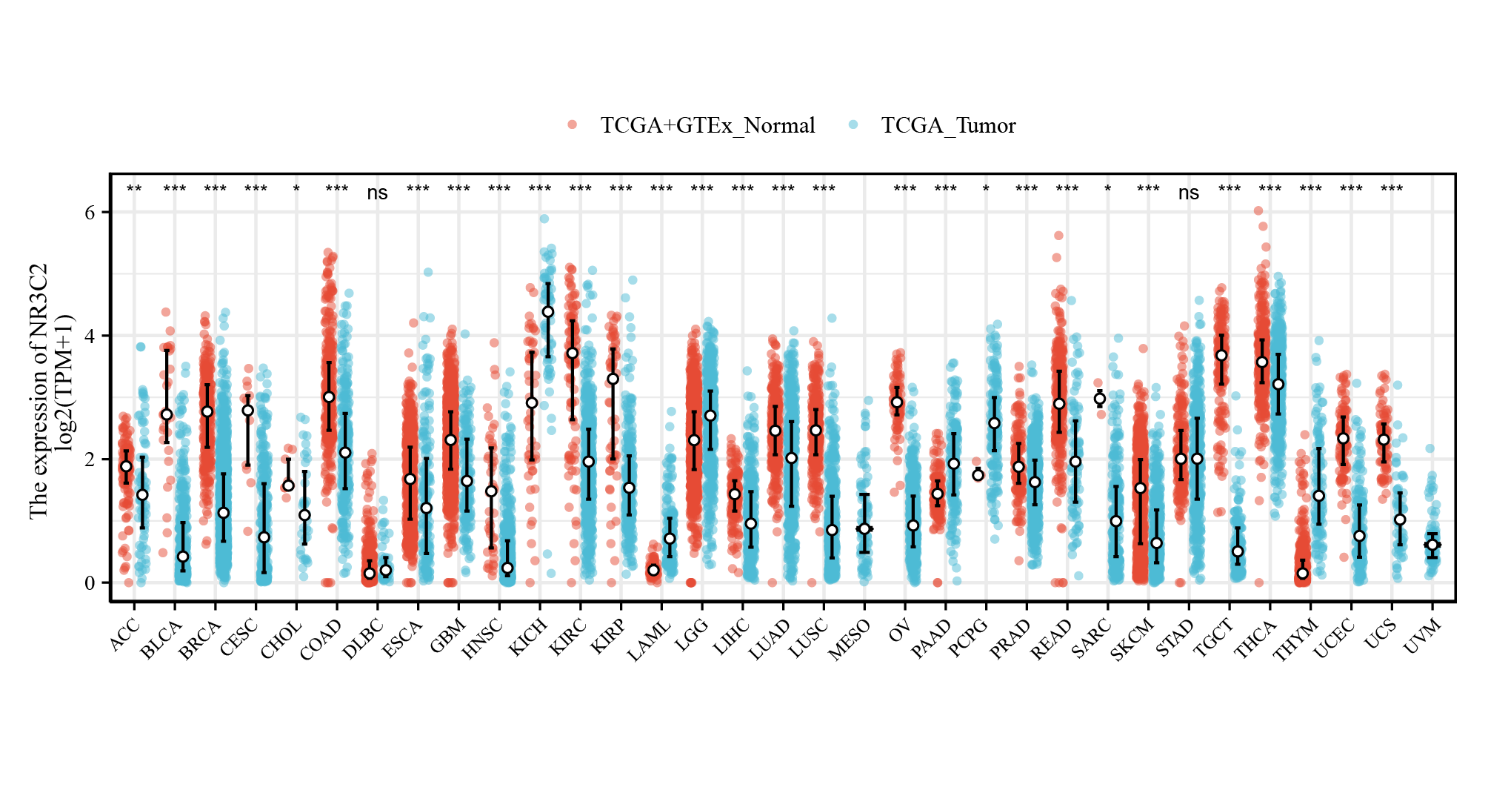


**Figure S1** Human NR3C2 expressions in pan-cancer (TCGA and GTEx databases). ACC, adrenocortical carcinoma; BLCA, bladder urothelial carcinoma; BRCA, breast invasive carcinoma; CESC, cervical squamous cell carcinoma and adenocarcinoma; CHOL, cholangiocarcinoma; COAD, colon cancer; ESCA, esophageal carcinoma; GBM, multiform glioma; HNSC, head and neck squamous cells carcinoma; KICH, renal chromophobe cell carcinoma; KIRC, renal clear cell carcinoma; KIRP, renal papillary cell carcinoma; LAML, acute myeloid leukemia; LGG, brain low-grade glioma; LIHC, hepatocellular carcinoma; LUAD, lung adenocarcinoma; Cancer LUSC, lung squamous cell carcinoma; OV, ovarian serous cystadenocarcinoma; PAAD, pancreatic cancer; PCPG, pheochromocytoma and paraganglioma; PRAD, prostate cancer; READ, rectal adenocarcinoma; SARC, sarcoma; SKCM, cutaneous melanoma; TGCT, testicular cancer; THCA, thyroid cancer; THYM, thymic cancer; UCEC, endometrial cancer; and UCS, uterine sarcoma; TCGA, The Cancer Genome Atlas; GTX, Genotype-Tissue Expression. * P<0.05, ** P<0.01, ***, P<0.001.


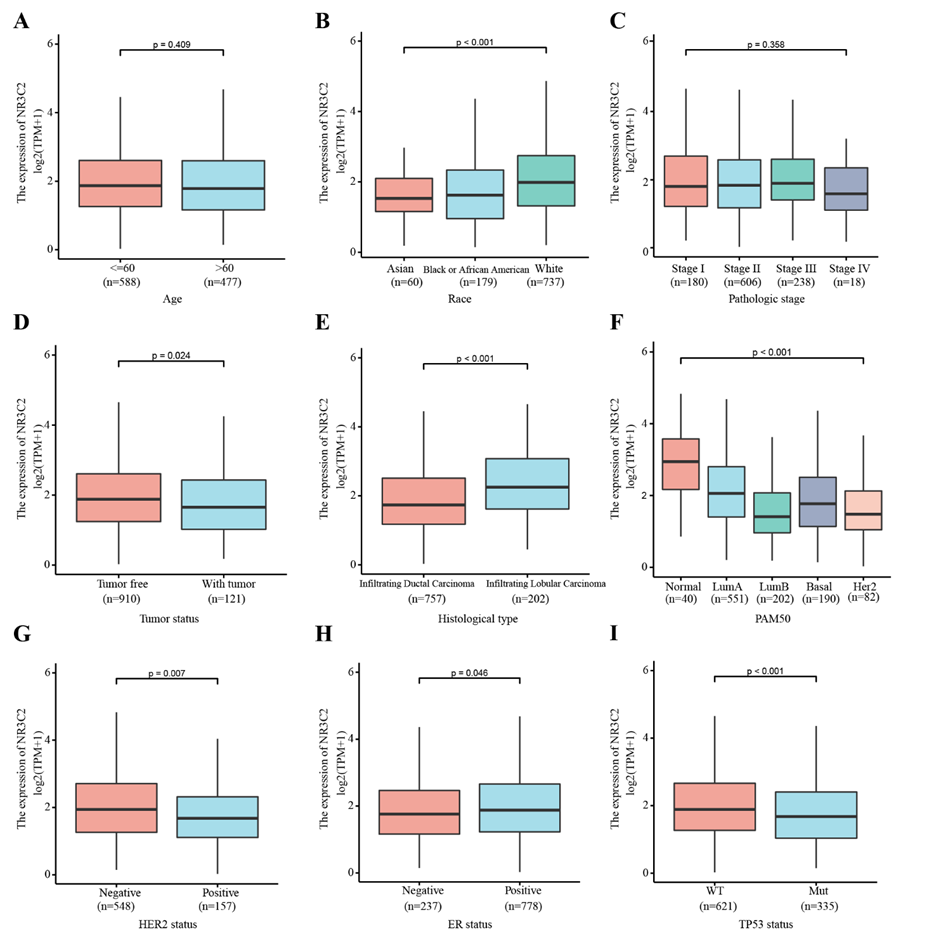


**Figure S2** NR3C2 expression in subgroups of patients with BRCA, stratified based on various clinicopathological characteristics including age, race, pathologic stage, tumor status, histological type, PAM50 and HER2/ER/TP53 status in the TCGA database **(A-I)**. * P< 0.05, ** P<0.01, *** P<0.001.


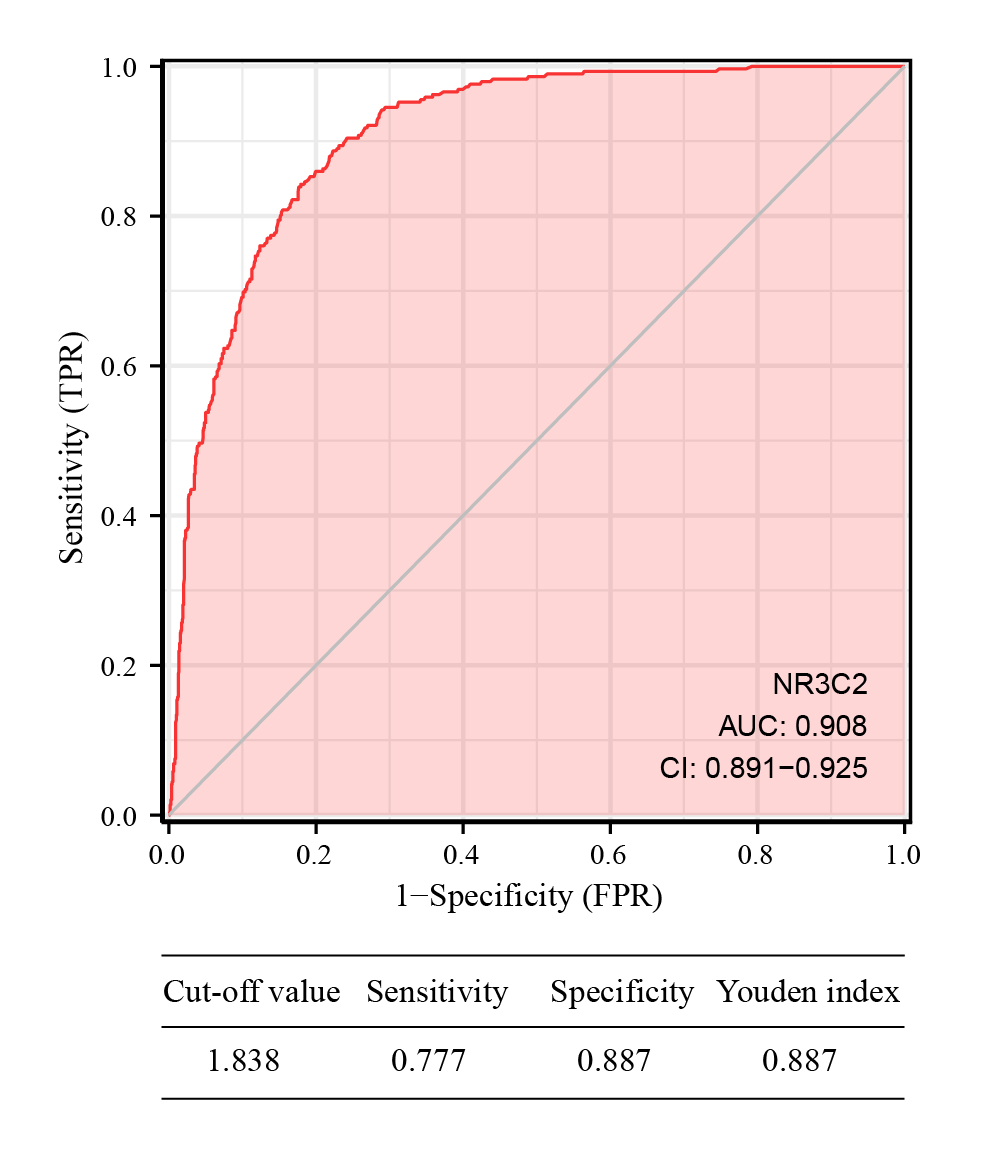


**Figure S3** Diagnostic efficacy of NR3C2 in breast invasive carcinoma evaluated by ROC curves. ROC, Receiver Operating Characteristic.


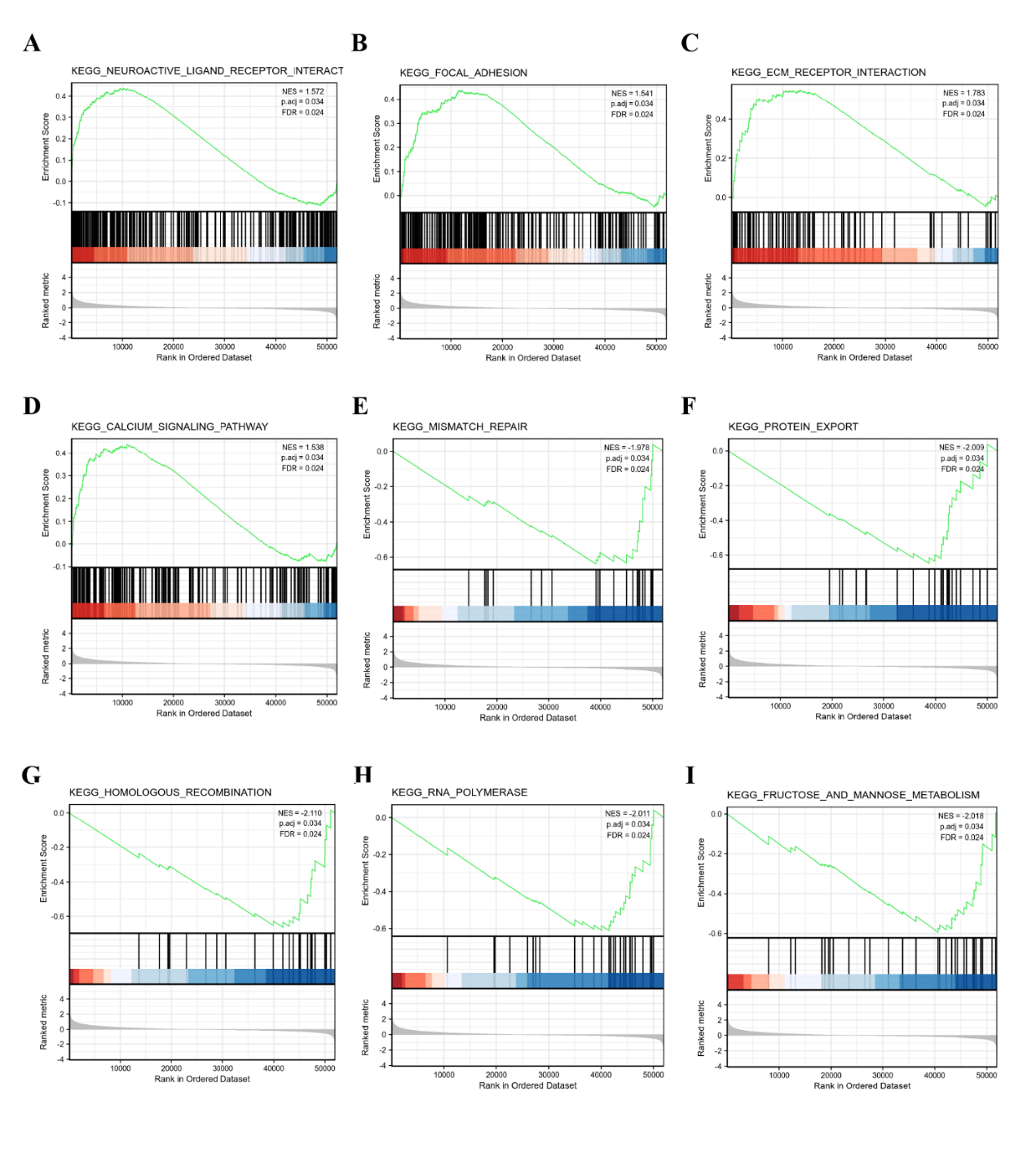


**Figure S4** Enrichment plots from the gene set enrichment analysis (GSEA) tool. Several pathways were differentially enriched in high NR3C2 expression phenotype of BRCA, including the neuroactive ligand receptor interact (A), focal adhesion (B), ECM receptor interaction (C), calcium signaling pathway (D), mismatch repair (E), protein export (F), homologous recombination (G), RNA polymerase (H) and fructose and mannose metabolism (I). The GSEA software was used to calculate the enrichment levels. GSEA, gene set enrichment analysis. ES, enrichment score; NES, normalized ES; ADJP-val, adjusted P-value.

# Supplementary Tables

**Table S1** NR3C2 expression was associated with several clinicopathological characteristics.

| Characteristics | N | Odds Ratio  (High- vs low-NR3C2 expression) | P value |
| --- | --- | --- | --- |
| Age (>60 vs. <=60) | 1065 | 0.90(0.70-1.14) | 0.370 |
| Race (Black or African American&Asian vs. White) | 976 | 0.46(0.34-0.61) | <0.001 |
| Anatomic neoplasm subdivisions (Right vs. Left) | 1065 | 1.17(0.92-1.48) | 0.209 |
| Menopause status (Pre&Peri vs. Post) | 956 | 1.00(0.76-1.33) | 0.974 |
| Pathologic stage (Stage II&Stage III&Stage IV vs. Stage I) | 1042 | 1.10(0.80-1.52) | 0.564 |
| T stage (T2&T3&T4 vs. T1) | 1062 | 0.86(0.66-1.14) | 0.294 |
| N stage (N1&N2&N3 vs. N0) | 1046 | 1.12(0.88-1.43) | 0.353 |
| M stage (M1 vs. M0) | 909 | 0.66(0.26-1.61) | 0.366 |
| Tumor status (With tumor vs. Tumor free) | 1031 | 0.69(0.47-1.00) | 0.053 |
| radiation therapy (Yes vs. No) | 972 | 1.17(0.91-1.51) | 0.217 |
| TP53 status (Mut vs. WT) | 956 | 0.64(0.49-0.83) | 0.001 |
| PIK3CA status (Mut vs. WT) | 956 | 1.00(0.76-1.31) | 0.990 |
| PAM50 (LumB&Her2&Basal vs. LumA) | 1025 | 0.42(0.32-0.54) | <0.001 |
| PR status (Positive vs. Negative) | 1012 | 1.17(0.90-1.53) | 0.230 |
| ER status (Positive vs. Negative) | 1015 | 1.32(0.99-1.77) | 0.062 |
| HER2 status (Positive vs. Negative) | 705 | 0.65(0.45-0.93) | 0.018 |
| Histological type (Infiltrating Ductal Carcinoma vs. Infiltrating Lobular Carcinoma) | 959 | 0.42(0.30-0.58) | <0.001 |

**Table S2** Univariate/multivariate Cox regression analysis on the progression-free survival in BRCA

| Characteristics | Total(N) | HR(95% CI) Univariate analysis | P value Univariate analysis | HR(95% CI) Multivariate analysis | P value Multivariate analysis |
| --- | --- | --- | --- | --- | --- |
| Age (>60 vs. <=60) | 1064 | 1.232(0.884-1.716) | 0.218 |  |  |
| Anatomic neoplasm subdivisions (Right vs. Left) | 1064 | 0.850(0.610-1.184) | 0.337 |  |  |
| Menopause status (Pre&Peri vs. Post) | 955 | 0.889(0.595-1.330) | 0.568 |  |  |
| Pathologic stage (Stage II&Stage III&Stage IV vs. Stage I) | 1041 | 2.214(1.293-3.792) | 0.004 | 1.141(0.402-3.235) | 0.805 |
| T stage (T2&T3&T4 vs. T1) | 1061 | 1.846(1.213-2.810) | 0.004 | 1.467(0.664-3.243) | 0.343 |
| N stage (N1&N2&N3 vs. N0) | 1045 | 2.250(1.559-3.245) | <0.001 | 1.846(1.155-2.951) | 0.010 |
| M stage (M1 vs. M0) | 909 | 8.288(4.812-14.275) | <0.001 | 5.342(2.753-10.365) | <0.001 |
| radiation therapy (Yes vs. No) | 971 | 0.885(0.620-1.264) | 0.502 |  |  |
| TP53 status (Mut vs. WT) | 955 | 1.258(0.879-1.802) | 0.210 |  |  |
| PIK3CA status (Mut vs. WT) | 955 | 0.838(0.565-1.245) | 0.382 |  |  |
| PAM50 (LumB&Her2&Basal vs. LumA) | 1024 | 1.458(1.040-2.042) | 0.029 | 0.771(0.466-1.277) | 0.313 |
| Race (Black or African American&Asian vs. White) | 975 | 1.179(0.796-1.747) | 0.410 |  |  |
| PR status (Positive vs. Negative) | 1011 | 0.567(0.405-0.795) | <0.001 | 0.522(0.287-0.951) | 0.034 |
| ER status (Positive vs. Negative) | 1014 | 0.599(0.419-0.857) | 0.005 | 0.776(0.417-1.442) | 0.422 |
| HER2 status (Positive vs. Negative) | 705 | 1.221(0.707-2.109) | 0.475 |  |  |
| Histological type (Infiltrating Ductal Carcinoma vs. Infiltrating Lobular Carcinoma) | 959 | 1.236(0.772-1.979) | 0.379 |  |  |
| NR3C2 (High vs. Low) | 1064 | 0.682(0.489-0.952) | 0.024 | 0.558(0.371-0.838) | 0.005 |

**Table S3** Univariate/multivariate Cox regression analysis on the disease-specific survival in BRCA

| Characteristics | Total(N) | HR(95% CI) Univariate analysis | P value Univariate analysis | HR(95% CI) Multivariate analysis | P value Multivariate analysis |
| --- | --- | --- | --- | --- | --- |
| Age (>60 vs. <=60) | 1045 | 1.418(0.913-2.201) | 0.120 |  |  |
| Anatomic neoplasm subdivisions (Right vs. Left) | 1045 | 0.797(0.513-1.239) | 0.314 |  |  |
| Menopause status (Pre&Peri vs. Post) | 945 | 0.628(0.344-1.147) | 0.130 |  |  |
| Pathologic stage (Stage II&Stage III&Stage IV vs. Stage I) | 1024 | 3.245(1.410-7.471) | 0.006 | 1.807(0.639-5.105) | 0.264 |
| T stage (T2&T3&T4 vs. T1) | 1042 | 1.700(0.983-2.940) | 0.058 |  |  |
| N stage (N1&N2&N3 vs. N0) | 1027 | 3.584(2.089-6.148) | <0.001 | 2.586(1.350-4.953) | 0.004 |
| M stage (M1 vs. M0) | 891 | 7.697(4.112-14.407) | <0.001 | 6.995(3.358-14.571) | <0.001 |
| radiation therapy (Yes vs. No) | 962 | 0.755(0.458-1.244) | 0.271 |  |  |
| TP53 status (Mut vs. WT) | 936 | 1.481(0.925-2.371) | 0.102 |  |  |
| PIK3CA status (Mut vs. WT) | 936 | 0.885(0.526-1.489) | 0.646 |  |  |
| PAM50 (LumB&Her2&Basal vs. LumA) | 1006 | 1.920(1.225-3.011) | 0.004 | 0.807(0.419-1.557) | 0.523 |
| Race (Black or African American&Asian vs. White) | 957 | 1.258(0.756-2.092) | 0.377 |  |  |
| PR status (Positive vs. Negative) | 993 | 0.529(0.336-0.833) | 0.006 | 0.645(0.274-1.519) | 0.316 |
| ER status (Positive vs. Negative) | 996 | 0.523(0.326-0.838) | 0.007 | 0.454(0.190-1.084) | 0.075 |
| HER2 status (Positive vs. Negative) | 695 | 1.481(0.740-2.965) | 0.267 |  |  |
| Histological type (Infiltrating Ductal Carcinoma vs. Infiltrating Lobular Carcinoma) | 941 | 2.002(0.957-4.188) | 0.065 |  |  |
| NR3C2 (High vs. Low) | 1045 | 0.570(0.363-0.895) | 0.015 | 0.443(0.259-0.758) | 0.003 |

**Table S4** Several significantly enriched GO terms in phenotype high.

| GO terms | ID | setSize | NES | P value | FDR |
| --- | --- | --- | --- | --- | --- |
| BP | Muscle system process | 450 | 1.656 | 0.001 | 0.079 |
|  | Regulation of membrane potential | 418 | 1.489 | 0.001 | 0.079 |
|  | Muscle tissue development | 395 | 1.486 | 0.001 | 0.079 |
|  | Regulation of vasculature development | 382 | 1.621 | 0.001 | 0.079 |
|  | Muscle contraction | 347 | 1.684 | 0.001 | 0.079 |
| CC | Receptor complex | 393 | 1.542 | 0.001 | 0.032 |
|  | Contractile fiber | 227 | 1.657 | 0.001 | 0.032 |
|  | Sarcolemma | 134 | 1.706 | 0.001 | 0.032 |
|  | I band | 135 | 1.752 | 0.001 | 0.032 |
|  | Basement membrane | 91 | 1.81 | 0.001 | 0.032 |
| MF | Extracellular matrix structural constituent | 160 | 1.828 | 0.001 | 0.056 |
|  | Peptide receptor activity | 151 | 1.642 | 0.001 | 0.056 |
|  | Integrin binding | 119 | 1.763 | 0.001 | 0.056 |
|  | Cytokine receptor activity | 95 | 1.78 | 0.001 | 0.056 |
|  | Extracellular matrix structural constituent conferring tensile strength | 41 | 1.926 | 0.001 | 0.056 |

BP: Biological process. CC: Cellular component. MF: Molecular function. NES: Normalized enrichment score; FDR: False discovery rate. P<0.05 was considered as significant.

**Table S5** TOP 20 KEGG pathways enriched in phenotype high.

| Terms | setSize | NES | P value | P.adjust | FDR |
| --- | --- | --- | --- | --- | --- |
| Neuroactive ligand receptor interaction | 268 | 1.572 | 0.001 | 0.034 | 0.024 |
| Focal adhesion | 197 | 1.541 | 0.001 | 0.034 | 0.024 |
| ECM receptor interaction | 83 | 1.783 | 0.001 | 0.034 | 0.024 |
| Calcium signaling pathway | 177 | 1.538 | 0.002 | 0.034 | 0.024 |
| Mismatch repair | 23 | -1.978 | 0.003 | 0.034 | 0.024 |
| Protein export | 23 | -2.009 | 0.003 | 0.034 | 0.024 |
| Homologous recombination | 26 | -2.11 | 0.003 | 0.034 | 0.024 |
| RNA polymerase | 29 | -2.011 | 0.003 | 0.034 | 0.024 |
| Fructose and mannose metabolism | 33 | -2.018 | 0.003 | 0.034 | 0.024 |
| Base excision repair | 34 | -2.341 | 0.004 | 0.034 | 0.024 |
| DNA replication | 36 | -2.497 | 0.004 | 0.034 | 0.024 |
| Nucleotide excision repair | 44 | -1.999 | 0.004 | 0.034 | 0.024 |
| Proteasome | 44 | -2.377 | 0.004 | 0.034 | 0.024 |
| Dilated cardiomyopathy | 90 | 1.704 | 0.004 | 0.034 | 0.024 |
| Aminoacyl TRNA biosynthesis | 41 | -1.968 | 0.004 | 0.034 | 0.024 |
| Glutathione metabolism | 49 | -1.974 | 0.004 | 0.034 | 0.024 |
| Vibrio cholerae infection | 53 | -1.819 | 0.004 | 0.034 | 0.024 |
| Pathogenic escherichia coli infection | 56 | -1.87 | 0.004 | 0.034 | 0.024 |
| Cardiac muscle contraction | 77 | -1.637 | 0.005 | 0.034 | 0.024 |
| Hypertrophic cardiomyopathy HCM | 83 | 1.645 | 0.005 | 0.034 | 0.024 |

NES: Normalized enrichment score; FDR: False discovery rate. Gene sets with p-adjust<0.05 was considered as significant.
